# Factors Associated with Chronic Low Back Pain in Hungary Based on the European Health Interview Surveys Conducted in 2009, 2014, and 2019: A Repeated Cross-Sectional Study

**DOI:** 10.3390/healthcare14091159

**Published:** 2026-04-25

**Authors:** Balázs Lukács, Amr Sayed Ghanem, Judit Molnár, Ilona Veres-Balajti, Attila Csaba Nagy

**Affiliations:** 1 Department of Physiotherapy, Institute of Health Sciences, Faculty of Health Sciences, University of Debrecen, 4028 Debrecen, Hungary; m.judit@etk.unideb.hu (J.M.); balajti.ilona@etk.unideb.hu (I.V.-B.); 2 Department of Epidemiology, Institute of Health Sciences, Faculty of Health Sciences, University of Debrecen, 4028 Debrecen, Hungary; aghanem@etk.unideb.hu (A.S.G.); nagy.attila@etk.unideb.hu (A.C.N.)

**Keywords:** low back pain, disability, European Health Interview Survey, chronic disease, sociodemographic

## Abstract

**Introduction**: Low back pain (LBP) is the leading cause of disability worldwide, with substantial variation in prevalence across regions. It is associated with a wide range of biophysical, psychological, social, and lifestyle factors, as well as comorbid conditions. Given its high impact, identifying population-level correlations of LBP is essential for informing prevention strategies. This study aimed to assess demographic, socioeconomic, lifestyle, and health-related factors associated with LBP in Hungary. **Methods**: A repeated cross-sectional analysis was conducted using secondary data from three waves of the European Health Interview Survey (EHIS) carried out in Hungary in 2009, 2014, and 2019. **Results**: The prevalence of LBP increased over the study period. Female sex, higher educational attainment, normal body mass index, non-smoking status, abstaining from alcohol, and good self-perceived health were associated with lower odds of LBP. In contrast, older age (≥65 years), unfavorable financial status, residence in socioeconomically disadvantaged regions, use of over-the-counter medications, and several chronic conditions were associated with higher odds. **Conclusions**: Reducing the impact of low back pain requires its integration into comprehensive public health frameworks that combine chronic disease management with consideration of socioeconomic inequalities at the population level.

## 1. Introduction

It is important to note that lower back pain is not a disease, but rather a symptom—pain that occurs in a specific area of the body, the cause of which is often unclear, and which is influenced by several closely interrelated factors. Global Burden of Disease studies define low back pain (LBP) as follows: “pain in the area on the posterior aspect of the body from the lower margin of the twelfth ribs to the lower gluteal folds with or without pain referred into one or both lower limbs that lasts for at least one day” [1].

Based on the duration of the pain, a distinction is made between acute (lasting up to 6 weeks), subacute (lasting between 7 and 12 weeks), and chronic (lasting longer than 12 weeks) types [2]. In addition, based on the cause of LBP, specific and nonspecific low back pain are differentiated. While specific low back pain is caused by a lesion of the musculoskeletal system or by an identified disease, the cause of nonspecific low back pain, which accounts for 90–95% of cases of low back pain, cannot be identified [3].

According to the Global Burden of Diseases, Injuries, and Risk Factors Study (GBD), LBP is the leading cause of disability in many countries [4]. According to that study published in 2023, the global number of LBP cases is projected to reach 619 million in 2020, rising to 843 million in 2050. With a standardized prevalence of 7460 per 100,000, the public health significance of LBP is demonstrated by the fact that low back pain is the main contributor to YLDs (years lived with disability) globally. There are significant geographical differences in prevalence (rate per 100,000 population), with the highest prevalence in Central (12,800; 11,500–14,400) and Eastern Europe (11,200; 10,100–12,500) and the lowest in East Asia (5430; 4870–6110). Our scientific study was motivated by the fact that Hungary has the highest incidence rate per 100,000 inhabitants in the world (14,000; 95% confidence interval: 12,600–15,500). Furthermore, it is important to note that in 2021, the age-standardized YLD rate for low back pain among adults aged 55 and older was the highest in the world in Hungary (3508.1; 95% CI: 2452.7–4772.7) [5,6]. Although there are significant differences between countries and regions in prevalence figures, LBP is an increasing pressure on health care systems in high-, middle-, and low-income countries.

Several non-modifiable (age, sex) and modifiable (smoking, physical activity, mental health) risk factors for the disorder have already been identified and could form the basis of future prevention programs. In a study published in 2024, Li investigated risk factors for LBP in the Chinese population and identified smoking, overweight (BMI ≥ 28 kg/m^2^), female sex, occupational exposure to vibration, overtime work, and lack of physical activity as risk factors [7]. Obesity, smoking, and strenuous physical work as risk factors and walking or cycling to work as protective factors were identified in a previous study focused on population of Finland [8]. In Hartvigsen et al.’s reviews, many other contributors to low back pain and disability have been identified, including biophysical (muscle size and composition), psychological (depression, anxiety, catastrophizing), social (low income and short education), lifestyle (BMI, smoking, physical activity), genetic factors, and comorbidities (vertebral fracture, axial spondyloarthritis, malignancy, infections, cauda equina syndrome) [9]. It is also important to note that extraordinary events, such as the COVID-19 pandemic and the associated lockdowns, also have an impact on the prevalence of LBP. Myalgia is a common symptom of COVID-19; in addition, changes in work conditions (home office), reduced physical activity, an increase in sedentary lifestyles, the resulting obesity, and negative emotional responses can all be associated with LBP [10]. As can be seen, a wide variety of risk and protective factors associated with LBP have been identified in recent years, but there is no data on which factors influence the development of LBP in Hungary.

Lower back pain is a public health problem that affects both the working-age population and the elderly. It has significant social and economic implications, as both its direct (healthcare) and indirect (absenteeism or reduced productivity) costs are high [1]. The impact of low back pain is independent of economic background; thus, it is a global problem that affects low-income, middle-income, and high-income countries as well [11,12]. Prevention and patient care play an important role in reducing the pressure on society; therefore, it is particularly important to identify the factors contributing to the development of LBP, which can help generate further scientific research that ultimately contributes to reducing the social problems caused by LBP through prevention and patient care. As mentioned above, there are significant differences in the prevalence and consequences of LBP among different countries; therefore, it is particularly important to emphasize national-level research into the factors influencing the development of LBP.

In this study, we hypothesized that prevalence of chronic low back pain in Hungary, using data from three waves of the Hungarian European Health Interview Survey affected by demographic, socioeconomic, and lifestyle factors and comorbid chronic diseases. Our repeated cross-sectional study was characterized by a large, nationally representative sample, offering externally valid findings on the broad inclusion of determinants of chronic low back pain.

## 2. Materials and Methods

### 2.1. Data Source

The European Health Interview Survey (EHIS) waves conducted in 2009, 2014, and 2019 provide nationally representative health-related data for the Hungarian population aged 15 years and older living in private households. The present study is a repeated cross-sectional analysis based on secondary data from these surveys, which employ a standardized, stratified sampling design to ensure population-level representativeness conducted using the STROBE guideline for observational studies [13]. The findings of this study should also be considered in light of the representativeness of the underlying data. The EHIS is based on a standardized, nationally representative sampling framework, ensuring that the results reflect the adult Hungarian population. While the analysis is country-specific, the use of a harmonized European survey instrument enhances comparability with other populations. As such, the observed patterns may be relevant for understanding low back pain within similar European and high-income settings, particularly in the context of shared demographic trends, socioeconomic structures, and chronic disease burden.

Data collection was harmonized across EU member states using a uniform questionnaire administered through a combination of electronic and face-to-face interviews, supporting comparability across survey waves. The analysis included all three Hungarian EHIS waves, comprising 5051 participants in 2009, 5826 in 2014, and 5603 in 2019, resulting in a pooled sample of 16,480 respondents.

Ethical approval was obtained from the Regional Ethics Committee of the University of Debrecen [5609–2020], and the study was conducted in accordance with the Declaration of Helsinki. Data were accessed between January and February 2024.

### 2.2. Variables of Interest

Socio-demographic variables included in the analysis were sex (male, female), age categories (15–34, 35–64, 65+), relationship status (single, in a relationship), educational attainment (primary, secondary, tertiary), employment status (employed, unemployed), area of residence (rural, urban), and geographical region as in NUTS2 (Central Hungary, Southern Great Plain, Southern Transdanubia, Northern Great Plain, Central Transdanubia, Northern Hungary, Western Transdanubia). Economic well-being was assessed through two measures: self-reported financial status (bad, average, good) and household income, stratified into quintiles ranging from lowest to highest income levels. Educational attainment was categorized into primary, secondary, and tertiary levels.

Lifestyle-related variables encompassed body mass index (BMI), classified as normal (BMI < 25) or overweight/obese (BMI ≥ 25), smoking status (smoker, non-smoker), and alcohol consumption (drinker, abstinent). Perceived health status was self-reported on a three-point ordinal scale (good, average, poor).

The study incorporated a wide range of chronic health conditions as explanatory variables, all of which were dichotomized (yes/no) to reflect their presence or absence. These conditions included the use of prescription drugs and over-the-counter (OTC) medications or supplements, asthma, bronchitis, acute myocardial infarction (AMI), coronary artery disease (CAD), stroke, arrhythmia, other heart diseases, hypertension, hypercholesterolemia, diabetes, arthrosis, chronic neck pain, allergies, peptic ulcers, chronic liver disease, cancer, migraine, depression, and other mental illnesses. Detailed description of how variables were classified has already been reported elsewhere [14,15,16].

Chronic low back pain used consistently as an outcome variable was defined based on a self-reported EHIS questionnaire item asking whether the respondent had experienced low back pain or other chronic back/spinal problems within the past 12 months. This measure reflects the presence of symptoms or conditions over a 12-month recall period.

### 2.3. Statistical Methods

The statistical analysis employed weighted proportions and Pearson’s chi-squared tests to describe associations between chronic low back pain and explanatory variables. These analyses were used for descriptive purposes only, while variable selection for multivariable modeling was guided primarily by epidemiological relevance and prior literature, rather than solely on statistical significance in bivariate testing.

Weighted binary multiple logistic regression models were constructed to estimate adjusted associations between the covariates and chronic low back pain. Model development followed a structured approach, beginning with a full model including sociodemographic, socioeconomic, lifestyle, and health-related variables considered relevant based on prior evidence. Alternative model specifications were evaluated during this process, including models incorporating survey year as an additional covariate. Model performance was compared using Akaike Information Criterion (AIC), Bayesian Information Criterion (BIC), and the area under the Receiver Operating Characteristic (ROC) curve (AUC), alongside overall classification performance. The final model was selected based on an optimal balance between goodness-of-fit, discriminative ability, and parsimony.

Model diagnostics were applied to assess calibration and overall fit, including the Hosmer–Lemeshow goodness-of-fit test. Discriminative performance was evaluated using ROC curve analysis, where the AUC provides a summary measure of the model’s ability to distinguish between individuals with and without chronic low back pain, with values closer to 1.0 indicating better discrimination [17,18].

Multicollinearity among independent variables was assessed using variance inflation factors, with a threshold of >5 indicating potential concern. No evidence of problematic multicollinearity was observed.

A complete case analysis approach was applied, whereby observations with missing values in any variables included in the multivariable models were excluded. This corresponds to listwise deletion during regression estimation in STATA.

All analyses were conducted using STATA IC Version 17.0, accounting for survey weights. Statistical significance was defined as a two-sided *p*-value < 0.05. Results from the final models are presented as adjusted odds ratios (ORs) with 95% confidence intervals (CIs).

## 3. Results

The EHIS included a total of 16,480 participants, with 5051 in 2009, 5826 in 2014, and 5603 in 2019. In 2009, out of 5050 respondents, 1621 reported chronic back pain. In 2014, 1782 out of 5825 respondents had chronic back pain. In 2019, 1832 out of 5575 respondents were reported to have chronic back pain. Overall, in the pooled sample of 16,450 respondents who answered the chronic back pain question, 5235 reported having chronic back pain.

### 3.1. Prevalence of Chronic Back Pain by Demographic and Socioeconomic Factors

In 2009, 45.65% of males and 54.35% of females reported chronic back pain (*p* = 0.368). In 2014, this increased to 44.58% for males and 55.42% for females (*p* = 0.021), and in 2019, to 43.59% for males and 56.41% for females (*p* = 0.001). Chronic back pain was significantly higher among participants aged 65+ in all years: 31.52% in 2009, 34.95% in 2014, and 31.97% in 2019 (*p* < 0.001). Single participants consistently reported lower prevalence (37.56% in 2009, 45.51% in 2014, 35.49% in 2019) compared to those in a relationship (62.44%, 54.49%, 64.51% respectively, *p* < 0.001). Participants with primary education had the highest prevalence: 62.03% in 2009, 61.11% in 2014, and 45.63% in 2019 (*p* < 0.001 for 2009 and 2014, *p* = 0.004 for 2019). Unemployed participants reported higher rates than employed ones in all years: 63.00% vs. 37.00% in 2009, 61.94% vs. 38.06% in 2014, and 51.33% vs. 48.67% in 2019 (*p* < 0.001). Rural residents had higher prevalence in 2009 and 2014 (30.08% vs. 69.92% and 31.64% vs. 68.36%, *p* < 0.001), but not in 2019 (28.83% vs. 71.17%, *p* = 0.362). Participants with bad financial status reported higher rates in all years: 37.68% in 2009, 28.84% in 2014, and 14.78% in 2019 (*p* < 0.001). Regional prevalence varied significantly, with Northern Great Plain reporting 16.03% in 2014 and 15.65% in the pooled sample, and Central Transdanubia 9.42% in 2014 and 9.88% in the pooled sample (*p* < 0.001) (Table 1).

### 3.2. Association of Chronic Back Pain with Lifestyle, Behavior, and Medication Factors

Chronic back pain was significantly associated with being overweight or obese across all years: 66.00% vs. 34.00% in 2009, 65.13% vs. 34.87% in 2014, and 66.85% vs. 33.15% in 2019 (*p* < 0.001 for all), as well as in the pooled sample (66.00% vs. 34.00%, *p* < 0.001). No significant difference was found between smokers and non-smokers in any year (*p* = 0.371 in 2009, *p* = 0.253 in 2014, *p* = 0.833 in 2019) or in the pooled sample (*p* = 0.982). Participants who abstained from alcohol reported higher rates of chronic back pain compared to drinkers in 2009 and 2014 (*p* = 0.09 and *p* < 0.001, respectively), with no significant difference in 2019 (*p* = 0.573) and a significant association in the pooled sample (34.30% vs. 65.70%, *p* < 0.001). Those with average self-perceived health consistently reported higher prevalence (43.09% in 2009, 43.01% in 2014, and 41.95% in 2019) compared to those with good or bad self-perceived health (*p* < 0.001 for all years), as well as in the pooled sample (42.69%, *p* < 0.001). Chronic back pain was more prevalent among participants with chronic diseases (96.47% in 2009, 71.14% in 2014, and 69.50% in 2019, *p* < 0.001 for all) and in the pooled sample (79.33%, *p* < 0.001). Participants who had not visited a dentist in more than a year reported higher prevalence (66.79% in 2009, 59.39% in 2014, and 57.46% in 2019, *p* < 0.001 for 2009 and 2014, *p* = 0.001 for 2019) and in the pooled sample (61.29%, *p* < 0.001). Those taking prescription drugs had higher prevalence (73.20% in 2009, 71.24% in 2014, and 66.57% in 2019, *p* < 0.001 for all) and in the pooled sample (70.36%, *p* < 0.001), as did those taking OTC drugs or supplements (43.41% in 2009, 54.39% in 2014, and 77.09% in 2019, *p* < 0.001 for all) and in the pooled sample (58.15%, *p* < 0.001) (Table 2).

### 3.3. Association of Chronic Back Pain with Different Medical Conditions

Chronic back pain was significantly associated with numerous medical conditions across the survey years. Participants with asthma reported higher rates of chronic back pain in 2009 (10.59% vs. 4.68%, *p* < 0.001), 2014 (8.13% vs. 3.50%, *p* < 0.001), and 2019 (7.97% vs. 3.49%, *p* < 0.001), as well as in the pooled sample (8.92% vs. 3.89%, *p* < 0.001). Bronchitis was also associated with higher prevalence in 2009 (12.36% vs. 3.65%, *p* < 0.001), 2014 (8.93% vs. 2.06%, *p* < 0.001), 2019 (7.83% vs. 2.45%, *p* < 0.001), and pooled (9.74% vs. 2.72%, *p* < 0.001). Higher rates were noted for AMI (2009: 7.76% vs. 2.65%, *p* < 0.001; 2014: 5.28% vs. 1.07%, *p* < 0.001; 2019: 3.95% vs. 1.41%, *p* < 0.001; pooled: 5.69% vs. 1.71%, *p* < 0.001) and CAD (2009: 14.81% vs. 3.80%, *p* < 0.001; 2014: 11.02% vs. 2.28%, *p* < 0.001; 2019: 6.97% vs. 2.07%, *p* < 0.001; pooled: 10.98% vs. 2.72%, *p* < 0.001). Hypertension was associated with increased prevalence (2009: 51.67% vs. 23.88%, *p* < 0.001; 2014: 52.92% vs. 22.77%, *p* < 0.001; 2019: 55.03% vs. 25.35%, *p* < 0.001; pooled: 49.88% vs. 23.99%, *p* < 0.001). Stroke showed higher prevalence in all years (2009: 4.03% vs. 2.20%, *p* < 0.001; 2014: 4.41% vs. 1.52%, *p* < 0.001; 2019: 3.01% vs. 1.55%, *p* < 0.001; pooled: 3.82% vs. 1.76%, *p* < 0.001). Participants with arthrosis consistently reported higher chronic back pain (2009: 53.30% vs. 10.14%, *p* < 0.001; 2014: 46.31% vs. 8.55%, *p* < 0.001; 2019: 34.84% vs. 10.48%, *p* < 0.001; pooled: 44.96% vs. 9.72%, *p* < 0.001). Chronic neck pain was significantly associated with chronic back pain (2009: 45.40% vs. 4.69%, *p* < 0.001; 2014: 37.56% vs. 4.15%, *p* < 0.001; 2019: 31.06% vs. 4.29%, *p* < 0.001; pooled: 38.08% vs. 4.38%, *p* < 0.001). Diabetes was also notable (2009: 13.31% vs. 6.07%, *p* < 0.001; 2014: 14.03% vs. 5.45%, *p* < 0.001; 2019: 12.42% vs. 7.25%, *p* < 0.001; pooled: 13.25% vs. 6.25%, *p* < 0.001). Additional significant associations were found with allergy (pooled: 22.26% vs. 14.37%, *p* < 0.001), peptic ulcer (pooled: 9.80% vs. 2.62%, *p* < 0.001), chronic liver disease (pooled: 1.67% vs. 0.35%, *p* < 0.001), cancer (pooled: 3.59% vs. 1.83%, *p* < 0.001), migraine (pooled: 24.77% vs. 9.37%, *p* < 0.001), depression (pooled: 10.45% vs. 2.63%, *p* < 0.001), mental illness (pooled: 8.94% vs. 3.30%, *p* < 0.001), hypercholesterolemia (pooled: 22.25% vs. 7.83%, *p* < 0.001), arrhythmia (pooled: 22.92% vs. 6.10%, *p* < 0.001), other heart disease (pooled: 6.23% vs. 1.94%, *p* < 0.001), and CVD (pooled: 31.29% vs. 10.25%, *p* < 0.001) (Table 3).

### 3.4. Weighted Multiple Logistic Regression Analysis of Factors Affecting Chronic Back Pain Prevalence

Females consistently had lower odds of chronic back pain compared to males, with a 28% decrease in 2009 (OR = 0.72, 95% CI: 0.60–0.86), 18% decrease in 2014 (OR = 0.82, 95% CI: 0.70–0.96), and 15% decrease in the pooled sample (OR = 0.85, 95% CI: 0.77–0.93). Participants aged 15–34 had significantly lower odds compared to those aged 65+, with reductions of 45% in 2009 (OR = 0.55, 95% CI: 0.39–0.77), 41% in 2014 (OR = 0.59, 95% CI: 0.44–0.78), and 39% in the pooled data (OR = 0.61, 95% CI: 0.51–0.72).

Being in a relationship increased the odds of chronic back pain, with a 21% increase in 2009 (OR = 1.21, 95% CI: 1.00–1.46), 28% in 2014 (OR = 1.28, 95% CI: 1.09–1.52), and 26% in the pooled sample (OR = 1.26, 95% CI: 1.14–1.38). Participants from the Southern Great Plain had higher odds compared to participants residing in central Hungary in 2009 (OR = 1.36, 95% CI: 1.03–1.78). Tertiary education was associated with lower odds in 2014 (OR = 0.73, 95% CI: 0.58–0.92) and in the pooled data (OR = 0.84, 95% CI: 0.72–0.96).

Those having bad financial status showing 30% higher odds in 2009 (OR = 1.30, 95% CI: 1.07–1.58) and 15% higher odds in the pooled sample (OR = 1.15, 95% CI: 1.02–1.29). Normal BMI was associated with lower odds in the pooled sample (OR = 0.85, 95% CI: 0.77–0.93). Non-smokers had consistently lower odds, with 26% lower odds in 2014 (OR = 0.74, 95% CI: 0.63–0.88) and 19% lower odds in the pooled data (OR = 0.81, 95% CI: 0.73–0.90). Abstaining from alcohol was associated with lower odds in 2009 (OR = 0.80, 95% CI: 0.67–0.95), 2019 (OR = 0.82, 95% CI: 0.69–0.98), and the pooled sample (OR = 0.84, 95% CI: 0.76–0.93).

Self-perceived health was a strong predictor, with good self-perceived health reducing odds by 46% in 2009 (OR = 0.54, 95% CI: 0.44–0.66), 49% in 2014 (OR = 0.51, 95% CI: 0.42–0.61), and 47% in the pooled data (OR = 0.53, 95% CI: 0.48–0.60). Conversely, bad self-perceived health increased the odds in 2009 (OR = 1.42, 95% CI: 1.08–1.88) and in the pooled sample (OR = 1.29, 95% CI: 1.11–1.51).

Taking OTC drugs or supplements was associated with higher odds, with a 36% increase in 2009 (OR = 1.36, 95% CI: 1.13–1.63), 23% in 2014 (OR = 1.23, 95% CI: 1.06–1.43), and 31% in the pooled sample (OR = 1.31, 95% CI: 1.20–1.44). Taking prescription drugs also increased the odds by 16% in the pooled data (OR = 1.16, 95% CI: 1.03–1.30).

Several medical conditions were significant predictors of chronic back pain. Arthrosis showed a consistent and significant increase in odds: 261% in 2009 (OR = 3.61, 95% CI: 2.94–4.42), 192% in 2014 (OR = 2.92, 95% CI: 2.40–3.54), and 189% in the pooled sample (OR = 2.89, 95% CI: 2.59–3.24). Chronic neck pain had even higher increases: 498% in 2009 (OR = 5.98, 95% CI: 4.70–7.61), 443% in 2014 (OR = 5.43, 95% CI: 4.34–6.79), and 446% in the pooled data (OR = 5.46, 95% CI: 4.78–6.23). Allergy also showed increased odds, with 35% in 2009 (OR = 1.35, 95% CI: 1.07–1.71), 40% in 2014 (OR = 1.40, 95% CI: 1.13–1.72), and 38% in the pooled sample (OR = 1.38, 95% CI: 1.22–1.56). Other notable increases were seen with arrhythmia, hypertension, hypercholesterolemia, peptic ulcer, and migraine across the years and in the pooled data.

Having cancer was associated with significantly lower odds of chronic back pain, with a 42% reduction in 2009 (OR = 0.58, 95% CI: 0.37–0.88) and 33% reduction in the pooled data (OR = 0.67, 95% CI: 0.51–0.88). Having suffered a stroke was also associated with lower odds in the pooled data (OR = 0.69, 95% CI: 0.53–0.90) (Table 4).

## 4. Discussion

Our study highlights the multifactorial nature of low back pain (LBP) in the adult Hungarian population, as identified through the European Health Interview Survey (EHIS). The results indicate that both modifiable factors—such as lifestyle characteristics, body mass index, smoking habits—and non-modifiable factors, including sex and age, are associated with the development and persistence of LBP. These findings are consistent with the previous studies, which highlight the complex interplay among demographic, socioeconomic, lifestyle, and health factors underlying chronic LBP.

Regarding demographic data, 2009, 2014, and pooled data of EHIS showed that female correlated with lower odds and individuals aged 65+ associated with higher odds for LBP compared to those aged 15–34. According to the “Global Burden of Disease 2010” study, like our study, the point prevalence of LBP was higher among males than among females [1]; in contrast, later GBD studies indicate a higher prevalence among females [4]. We believe that the differences in point prevalence are not due to changes in the development of back pain but rather can be explained by differences in data collection and statistical methods, such as crude and adjusted differences. According to Felício et al. and Bento et al., the sex difference can be associated with differences in lifestyle, as well as environmental and socioeconomic factors such as confounding differences [19,20]. The prevalence of LBP differs across countries, which can be attributed to variations in socioeconomic and environmental factors. We believe that differences in psychological and sociocultural factors (men are more likely to perform heavy physical work) and lifestyle factors (men are more likely to smoke) among countries may explain why the prevalence of LBP was higher among males in our study [21,22].

Hartvigsen and colleagues also confirmed the link between aging and LBP; according to their study, the prevalence of LBP increases in middle age, and activity limitations due to LBP continue to rise with aging [9]. Furthermore, the 2021 Global Burden of Disease Study found that, globally, the peak incidence of lower back pain occurs around the age of 85 for both males and females [5]. According to Felíció et al., there is only limited evidence to support the idea that comorbidities, poor health, and a history of back pain are risk factors for low back pain; however, the prevalence of each of these factors increases with age [19]. In our view, it is a well-known fact that the incidence of chronic diseases (musculoskeletal disorders, cardiovascular diseases, and cancers) and psychosocial factors (sedentary lifestyle, depression) increase with aging; these factors, whether individually or in combination, can be linked to low back pain in older adults.

Our research also demonstrated the effect of socio-economic factors, showing that participants who were in a poor financial situation or who lived in a socio-economically deprived region (the Southern Great Plain region of Hungary) were associated with higher odds of LBP. Furthermore, this study also suggests a link between level of education—particularly tertiary education—and lower back pain. It is a well-known among public health professionals that various socioeconomic factors (education, occupation, and income) influence the development and progression of chronic diseases, and thereby also affect health status. The reason for this is that, according to the accepted model, people with higher socioeconomic status have higher incomes and easier access to healthcare services, possess more information about health, lead healthier lifestyles, are less exposed to environmental risk factors, and have greater influence over decisions affecting their health. This is supported by earlier studies conducted in Japan, Germany, and South Korea that suggest that the prevalence of LBP is higher among older adults with lower educational levels and lower incomes [23] and that health insurance status and the use of health services are independently associated with severe LBP [24,25].

According to our statistical analysis, LBP was also associated with lifestyle: normal BMI, non-smoking habits, and abstaining from alcohol correlated with lower odds of LBP. In previous studies, consistent with our findings, Li et al. found that smoking and a BMI ≥ 28 kg/m^2^ might be risk factors for LBP in the Chinese population [7] and Siri et al. also concluded that both current and former smokers have a higher prevalence and incidence of low back pain than those who have never smoked [26]. In contrast to our results, two previous studies suggest that smoking has no significant, only modest effect on future LBP [8,9]. In our model, smoking is associated with higher odds of LBP through several mechanisms, and these pathological processes can occur simultaneously and amplify each other’s effects. For example, smoking impairs blood perfusion in the blood vessels, causing poor circulation and hypoxia, and increases the risk of systemic inflammation, which can trigger pain. Moreover, smokers’ lower physical activity levels and postural disorders due to weak core muscles may also contribute to the LBP [27,28,29]. It is known that there is a negative correlation between BMI and physical activity; thus, the lower levels of physical activity observed among smokers can be associated with higher odds of BMI, which in turn is associated with higher odds of LBP [30,31,32]. Based on the above, low physical activity as a lifestyle factor is associated with a higher odds of low back pain, while increased physical activity is associated with a lower odd [33,34,35,36].

Based on EHIS data, good self-perceived health was associated with lower odds of LBP. In previous studies it was suggested that poor self-rated health and depressive symptoms were a risk factor for future LBP [37,38,39]. The lifestyles of those who rate their health as poor are often characterized by factors (physical inactivity, obesity, smoking, anxiety, depression) that, as discussed above, are associated with higher odds of LBP; thus, bad self-perceived health is thus linked to the development of LBP through other factors.

Our results suggested that taking over-the-counter medicines or dietary supplements and taking prescription medicines were associated with higher odds of LBP. Several explanations for this finding are possible. On the one hand, the use of prescription drugs or a combination of several drugs is typical of people in poor general health diagnosed with one or more chronic diseases. On the other hand, several co-morbid conditions have been identified to be associated with LBP, such as cardiovascular, rheumatologic, diabetes, malignancy, gynecologic, nephrology and urologic diseases [40,41,42,43,44,45,46,47,48,49,50,51,52].

Associations between LBP and comorbid conditions were also evaluated in our study. These results are supported by Australian and German surveys have shown that most patients have other comorbid conditions associated with back pain [40,41]. It is also important to consider that LBP can occur as a side effect of medications used to treat chronic conditions, so chronic diseases can be associated with higher odds of LBP both on their own and through medication. This concept is supported by two previous studies that found a link between the statin (management of hypercholesterolemia) and verapamil (management of hypertension) medications and low back pain [53,54].

Our study suggests that pre-existing musculoskeletal conditions (osteoarthritis and chronic neck pain) were associated with higher odds of LBP which can be explained by the fact that similar lifestyle factors are present in other musculoskeletal disorders (physical inactivity, stress) as in lower back pain. Furthermore, since the spine and its associate muscles form a functional unit, structural and functional disorders in the cervical region led to changes in the lower regions of the spine after a longer period. In addition, damage to the vascular system (e.g., atherosclerosis) can negatively affect the blood vessels that supply the spine, and the presence of ongoing chronic inflammation in the body, a common consequence of cardiovascular diseases, reduces the spine’s supply of nutrients and oxygen, as well as its ability to regenerate, leading to structural damage to the spine, the development of ischemia, and ultimately, lower back pain.

Regarding cancers, although it is not one of the most important symptoms, back pain can occur with certain tumours. In the previous studies, chronic back pain has been associated with colon, ovarian, or prostate cancer [55,56]. On the one hand, certain tumours can metastasize to the bone, where they reduce the bone’s mechanical strength, leading to pathological fractures and, consequently, pain; on the other hand, as tumours grow, they can also exert pressure on the nerves and the spinal cord, which can likewise cause pain in the affected region.

## 5. Strengths and Limitations

Our study has limitations. Our study was a repeated cross-sectional study; therefore, causal relationships cannot be established, and data collection based on self-reporting may cause recall bias. In addition, it should be noted that the duration and severity of chronic diseases are not included in our analysis, as these data were not available in the surveys. However, the study also has several strengths, including a reliable sampling methodology and comprehensive data collection and processing protocols managed by Eurostat and the Hungarian Central Statistical Office. The data from the three waves of the European Health Interview Survey (EHIS) conducted in Hungary in 2009, 2014, and 2019 are representative of the Hungarian adult population. In addition, the study used a standardized questionnaire that was applied in all European countries during three consecutive periods. External validity is high due to the representative sample, and because of the three measurement points (2009, 2014, 2019), the data can also be generalized over time; the large sample size reduces the likelihood of sampling bias, but causal relationships can only be examined to a limited extent.

## 6. Conclusions

The results indicate that low back pain is embedded within broader patterns of multimorbidity and socioeconomic inequality in the Hungarian population. Addressing its burden requires integration into comprehensive public health strategies that extend beyond individual-level risk factors to include systemic and population-level determinants, with particular emphasis on approaches that account for the complex interplay between social and clinical factors.

## Figures and Tables

**Table 1:** Distribution of chronic low back pain status across demographic and socioeconomic characteristics.

Characteristics		Chronic Back Pain											
		2009			2014			2019			Pooled		
		No (n, %)	Yes (n, %)	*p*-Value	No (n, %)	Yes (n, %)	*p*-Value	No (n, %)	Yes (n, %)	*p*-Value	No (n, %)	Yes (n, %)	*p*-Value
Year	2009										3429 (33.69%)	1621 (34.14%)	0.539
	2014										4043 (33.62%)	1782 (32.70%)	
	2019										3743 (32.69%)	1832 (33.16%)	
Sex	Male	1583 (47.08%)	714 (45.65%)	0.368	1925 (47.96%)	775 (44.58%)	**0.021**	1781 (48.79%)	776 (43.59%)	**0.001**	5289 (47.93%)	2265 (44.62%)	**<0.001**
	Female	1846 (52.92%)	907 (54.35%)		2118 (52.04%)	1007 (55.42%)		1962 (51.21%)	1056 (56.41%)		5926 (52.07%)	2970 (55.38%)	
Age categories	65+	532 (15.18%)	514 (31.52%)	**<0.001**	587 (14.42%)	629 (34.95%)	**<0.001**	913 (19.03%)	708 (31.97%)	**<0.001**	2032 (16.18%)	1851 (32.79%)	**<0.001**
	15 to 34	1319 (40.05%)	153 (10.31%)		1488 (35.47%)	219 (12.31%)		1060 (32.64%)	211 (15.00%)		3867 (36.09%)	583 (12.52%)	
	35 to 64	1578 (44.77%)	954 (58.17%)		1968 (50.10%)	934 (52.74%)		1770 (48.33%)	913 (53.03%)		5316 (47.73%)	2801 (54.69%)	
Relationship status	Single	1782 (50.42%)	650 (37.56%)	**<0.001**	2249 (54.88%)	813 (45.51%)	**<0.001**	1644 (44.19%)	679 (35.49%)	**<0.001**	5675 (49.93%)	2142 (39.50%)	**<0.001**
	In a relationship	1642 (49.58%)	970 (62.44%)		1790 (45.12%)	969 (54.49%)		2006 (55.81%)	1121 (64.51%)		5438 (50.07%)	3060 (60.50%)	
Education	Primary	1744 (49.34%)	1022 (62.03%)	**<0.001**	1859 (44.76%)	1114 (61.11%)	**<0.001**	1608 (40.55%)	889 (45.63%)	**0.004**	5211 (44.92%)	3025 (56.28%)	**<0.001**
	Secondary	1099 (32.74%)	443 (27.61%)		1316 (32.64%)	428 (24.69%)		1264 (34.58%)	568 (31.71%)		3679 (33.31%)	1439 (28.01%)	
	Tertiary	584 (17.92%)	153 (10.36%)		867 (22.60%)	240 (14.20%)		871 (24.88%)	375 (22.66%)		2322 (21.77%)	768 (15.70%)	
Employment	Unemployed	1732 (49.26%)	1037 (63.00%)	**<0.001**	1827 (44.24%)	1118 (61.94%)	**<0.001**	1746 (42.32%)	1039 (51.33%)	**<0.001**	5305 (45.31%)	3194 (58.78%)	**<0.001**
	Employed	1697 (50.74%)	584 (37.00%)		2211 (55.76%)	663 (38.06%)		1997 (57.68%)	793 (48.67%)		5905 (54.69%)	2040 (41.22%)	
Area of residence	Rural	948 (24.74%)	530 (30.08%)	**<0.001**	1183 (27.43%)	610 (31.64%)	**0.001**	1240 (30.05%)	563 (28.83%)	0.362	3371 (27.38%)	1703 (30.18%)	**<0.001**
	Urban	2481 (75.26%)	1091 (69.92%)		2860 (72.57%)	1172 (68.36%)		2503 (69.95%)	1269 (71.17%)		7844 (72.62%)	3532 (69.82%)	
Regions	Central-Hungary	858 (30.67%)	374 (27.13%)	**0.001**	1060 (31.09%)	429 (27.71%)	**<0.001**	1058 (30.85%)	531 (30.72%)	0.197	2976 (30.87%)	1334 (28.51%)	**<0.001**
	Southern Great Plain	459 (12.10%)	269 (15.75%)		551 (12.59%)	260 (13.89%)		476 (13.10%)	214 (12.13%)		1486 (12.59%)	743 (13.94%)	
	Southern Transdanubia	320 (9.07%)	180 (10.51%)		375 (8.68%)	196 (10.75%)		376 (9.25%)	153 (8.64%)		1071 (9.00%)	529 (9.97%)	
	Northern Great Plain	549 (14.23%)	271 (15.56%)		656 (14.29%)	323 (16.03%)		566 (14.36%)	301 (15.38%)		1771 (14.29%)	895 (15.65%)	
	Central Transdanubia	402 (11.44%)	154 (9.92%)		508 (11.57%)	173 (9.42%)		428 (11.25%)	189 (10.30%)		1338 (11.42%)	516 (9.88%)	
	Northern Hungary	453 (11.92%)	219 (12.18%)		449 (11.04%)	247 (13.46%)		445 (10.66%)	252 (12.96%)		1347 (11.21%)	718 (12.85%)	
	Western Transdanubia	388 (10.58%)	154 (8.94%)		444 (10.74%)	154 (8.74%)		394 (10.54%)	192 (9.88%)		1226 (10.62%)	500 (9.19%)	
Financial status	Average	2033 (60.17%)	877 (54.55%)	**<0.001**	2294 (56.72%)	1025 (57.58%)	**<0.001**	2030 (53.98%)	1094 (60.15%)	**<0.001**	6357 (57.00%)	2996 (57.38%)	**<0.001**
	Good	512 (15.28%)	121 (7.77%)		988 (25.17%)	238 (13.59%)		1244 (35.98%)	425 (25.07%)		2744 (25.32%)	784 (15.35%)	
	Bad	854 (24.55%)	614 (37.68%)		724 (18.10%)	509 (28.84%)		385 (10.04%)	275 (14.78%)		1963 (17.68%)	1398 (27.27%)	
Income quintiles	First	688 (19.85%)	316 (20.36%)	0.088	652 (15.68%)	513 (28.15%)	**<0.001**	768 (19.91%)	384 (20.54%)	0.167	2108 (18.47%)	1213 (22.97%)	**<0.001**
	Second	685 (20.43%)	308 (19.10%)		749 (17.99%)	415 (22.53%)		752 (19.43%)	417 (21.29%)		2186 (19.28%)	1140 (20.95%)	
	Third	650 (19.52%)	339 (20.98%)		896 (21.79%)	361 (20.04%)		753 (19.87%)	379 (20.19%)		2299 (20.40%)	1079 (20.41%)	
	Fourth	690 (19.44%)	362 (21.55%)		794 (19.88%)	260 (15.18%)		851 (22.54%)	410 (22.35%)		2335 (20.60%)	1032 (19.73%)	
	Fifth	716 (20.76%)	296 (18.01%)		952 (24.65%)	233 (14.11%)		619 (18.25%)	242 (15.62%)		2287 (21.25%)	771 (15.94%)	

Bold values indicate statistical significance (*p* < 0.05) based on weighted Pearson’s chi-squared test. Values are presented as weighted column percentages; counts are unweighted.

**Table 2:** Distribution of chronic low back pain status across lifestyle, behavioral, and medication-related factors.

Characteristics		Chronic Back Pain											
		2009			2014			2019			Pooled		
		No (n, %)	Yes (n, %)	*p*-Value	No (n, %)	Yes (n, %)	*p*-Value	No (n, %)	Yes (n, %)	*p*-Value	No (n, %)	Yes (n, %)	*p*-Value
BMI	Overweight/Obese	1671 (48.12%)	1070 (66.00%)	**<0.001**	1977 (49.11%)	1149 (65.13%)	**<0.001**	2084 (54.21%)	1236 (66.85%)	**<0.001**	5732 (50.44%)	3455 (66.00%)	**<0.001**
	Normal	1748 (51.88%)	548 (34.00%)		2059 (50.89%)	616 (34.87%)		1621 (45.79%)	578 (33.15%)		5428 (49.56%)	1742 (34.00%)	
Smoking	Smoker	1045 (31.85%)	478 (30.52%)	0.371	1103 (27.05%)	507 (28.55%)	0.253	1041 (28.13%)	491 (27.84%)	0.833	3189 (28.99%)	1476 (28.97%)	0.982
	Non-smoker	2285 (68.15%)	1102 (69.48%)		2932 (72.95%)	1275 (71.45%)		2702 (71.87%)	1341 (72.16%)		7919 (71.01%)	3718 (71.03%)	
Alcohol	Drinker	2120 (63.82%)	956 (61.24%)	0.09	2890 (72.29%)	1135 (65.25%)	**<0.001**	2619 (71.43%)	1263 (70.67%)	0.573	7629 (69.18%)	3354 (65.70%)	**<0.001**
	Abstinent	1270 (36.18%)	642 (38.76%)		1142 (27.71%)	645 (34.75%)		1124 (28.57%)	569 (29.33%)		3536 (30.82%)	1856 (34.30%)	
Self-perceived health	Average	907 (25.74%)	700 (43.09%)	**<0.001**	840 (20.80%)	777 (43.01%)	**<0.001**	955 (22.83%)	800 (41.95%)	**<0.001**	2702 (23.12%)	2277 (42.69%)	**<0.001**
	Good	2270 (67.33%)	396 (25.29%)		2985 (74.02%)	561 (32.45%)		2538 (71.78%)	640 (39.13%)		7793 (71.03%)	1597 (32.20%)	
	Bad	251 (6.93%)	525 (31.62%)		218 (5.19%)	444 (24.54%)		237 (5.39%)	382 (18.92%)		706 (5.84%)	1351 (25.11%)	
Chronic disease	No	1469 (43.47%)	57 (3.53%)	**<0.001**	2679 (66.25%)	500 (28.86%)	**<0.001**	2188 (62.16%)	497 (30.50%)	**<0.001**	6336 (57.21%)	1054 (20.67%)	**<0.001**
	Yes	1957 (56.53%)	1564 (96.47%)		1362 (33.75%)	1282 (71.14%)		1497 (37.84%)	1291 (69.50%)		4816 (42.79%)	4137 (79.33%)	
Last dental visit	More than a year ago	2060 (59.87%)	1094 (66.79%)	**<0.001**	2090 (51.71%)	1073 (59.39%)	**<0.001**	2029 (52.21%)	1072 (57.46%)	**0.001**	6179 (54.63%)	3239 (61.29%)	**<0.001**
	Within the last year	1368 (40.13%)	524 (33.21%)		1948 (48.29%)	703 (40.61%)		1674 (47.79%)	731 (42.54%)		4990 (45.37%)	1958 (38.71%)	
Taking prescription drugs	No	1906 (56.55%)	424 (26.80%)	**<0.001**	2412 (59.53%)	501 (28.76%)	**<0.001**	1971 (56.35%)	532 (33.43%)	**<0.001**	6289 (57.49%)	1457 (29.64%)	**<0.001**
	Yes	1523 (43.45%)	1196 (73.20%)		1630 (40.47%)	1281 (71.24%)		1756 (43.65%)	1294 (66.57%)		4909 (42.51%)	3771 (70.36%)	
Taking OTC drugs or supplements	No	2281 (65.92%)	938 (56.59%)	**<0.001**	2291 (55.80%)	822 (45.61%)	**<0.001**	762 (18.72%)	446 (22.91%)	**<0.001**	5334 (47.13%)	2206 (41.85%)	**<0.001**
	Yes	1147 (34.08%)	682 (43.41%)		1751 (44.20%)	960 (54.39%)		2965 (81.28%)	1380 (77.09%)		5863 (52.87%)	3022 (58.15%)	

Bold values indicate statistical significance (*p* < 0.05) based on weighted Pearson’s chi-squared test. Values are presented as weighted column percentages; counts are unweighted. Abbreviations: BMI, body mass index; OTC, over the counter.

**Table 3:** Distribution of chronic low back pain status across medical conditions.

Characteristics		Chronic Back Pain											
		2009			2014			2019			Pooled		
		No (n, %)	Yes (n, %)	*p*-Value	No (n, %)	Yes (n, %)	*p*-Value	No (n, %)	Yes (n, %)	*p*-Value	No (n, %)	Yes (n, %)	*p*-Value
Asthma	No	3272 (95.32%)	1451 (89.41%)	**<0.001**	3894 (96.50%)	1631 (91.87%)	**<0.001**	3594 (96.51%)	1675 (92.03%)	**<0.001**	10,760 (96.11%)	4757 (91.08%)	**<0.001**
	Yes	157 (4.68%)	170 (10.59%)		148 (3.50%)	151 (8.13%)		139 (3.49%)	147 (7.97%)		444 (3.89%)	468 (8.92%)	
Bronchitis	No	3308 (96.35%)	1416 (87.64%)	**<0.001**	3958 (97.94%)	1620 (91.07%)	**<0.001**	3635 (97.55%)	1668 (92.17%)	**<0.001**	10,901 (97.28%)	4704 (90.26%)	**<0.001**
	Yes	121 (3.65%)	203 (12.36%)		84 (2.06%)	162 (8.93%)		98 (2.45%)	147 (7.83%)		303 (2.72%)	512 (9.74%)	
AMI	No	3337 (97.35%)	1497 (92.24%)	**<0.001**	3998 (98.93%)	1684 (94.72%)	**<0.001**	3667 (98.59%)	1742 (96.05%)	**<0.001**	11,002 (98.29%)	4923 (94.31%)	**<0.001**
	Yes	92 (2.65%)	124 (7.76%)		45 (1.07%)	98 (5.28%)		68 (1.41%)	82 (3.95%)		205 (1.71%)	304 (5.69%)	
CAD	No	3297 (96.20%)	1375 (85.19%)	**<0.001**	3947 (97.72%)	1569 (88.98%)	**<0.001**	3636 (97.93%)	1668 (93.03%)	**<0.001**	10,880 (97.28%)	4612 (89.02%)	**<0.001**
	Yes	132 (3.80%)	245 (14.81%)		96 (2.28%)	212 (11.02%)		95 (2.07%)	148 (6.97%)		323 (2.72%)	605 (10.98%)	
Hypertension	No	2580 (76.12%)	766 (48.33%)	**<0.001**	3110 (77.23%)	833 (47.08%)	**<0.001**	2665 (74.65%)	915 (55.03%)	**<0.001**	8355 (76.01%)	2514 (50.12%)	**<0.001**
	Yes	848 (23.88%)	854 (51.67%)		933 (22.77%)	949 (52.92%)		1069 (25.35%)	895 (44.97%)		2850 (23.99%)	2698 (49.88%)	
Stroke	No	3352 (97.80%)	1555 (95.97%)	**<0.001**	3981 (98.48%)	1704 (95.59%)	**<0.001**	3665 (98.45%)	1763 (96.99%)	**<0.001**	10,998 (98.24%)	5022 (96.18%)	**<0.001**
	Yes	77 (2.20%)	66 (4.03%)		62 (1.52%)	78 (4.41%)		70 (1.55%)	60 (3.01%)		209 (1.76%)	204 (3.82%)	
Arthrosis	No	3079 (89.86%)	751 (46.70%)	**<0.001**	3689 (91.45%)	950 (53.69%)	**<0.001**	3286 (89.52%)	1105 (65.16%)	**<0.001**	10,054 (90.28%)	2806 (55.04%)	**<0.001**
	Yes	350 (10.14%)	868 (53.30%)		351 (8.55%)	831 (46.31%)		439 (10.48%)	691 (34.84%)		1140 (9.72%)	2390 (44.96%)	
Chronic neck pain	No	3265 (95.31%)	868 (54.60%)	**<0.001**	3873 (95.85%)	1101 (62.44%)	**<0.001**	3564 (95.71%)	1240 (68.94%)	**<0.001**	10,702 (95.62%)	3209 (61.92%)	**<0.001**
	Yes	164 (4.69%)	752 (45.40%)		170 (4.15%)	681 (37.56%)		173 (4.29%)	588 (31.06%)		507 (4.38%)	2021 (38.08%)	
Diabetes	No	3220 (93.93%)	1404 (86.69%)	**<0.001**	3818 (94.55%)	1531 (85.97%)	**<0.001**	3412 (92.75%)	1560 (87.58%)	**<0.001**	10,450 (93.75%)	4495 (86.75%)	**<0.001**
	Yes	209 (6.07%)	217 (13.31%)		225 (5.45%)	250 (14.03%)		308 (7.25%)	240 (12.42%)		742 (6.25%)	707 (13.25%)	
Allergy	No	2976 (86.15%)	1267 (78.08%)	**<0.001**	3533 (87.18%)	1435 (80.55%)	**<0.001**	3134 (83.49%)	1380 (74.58%)	**<0.001**	9643 (85.63%)	4082 (77.74%)	**<0.001**
	Yes	453 (13.85%)	354 (21.92%)		509 (12.82%)	347 (19.45%)		586 (16.51%)	429 (25.42%)		1548 (14.37%)	1130 (22.26%)	
Peptic ulcer	No	3272 (95.55%)	1350 (83.47%)	**<0.001**	3971 (98.17%)	1649 (92.64%)	**<0.001**	3672 (98.45%)	1710 (94.78%)	**<0.001**	10,915 (97.38%)	4709 (90.20%)	**<0.001**
	Yes	157 (4.45%)	271 (16.53%)		70 (1.83%)	133 (7.36%)		62 (1.55%)	100 (5.22%)		289 (2.62%)	504 (9.80%)	
Chronic liver disease	No	3408 (99.42%)	1578 (97.44%)	**<0.001**	4032 (99.76%)	1761 (98.91%)	**<0.001**	3724 (99.77%)	1780 (98.69%)	**<0.001**	11,164 (99.65%)	5119 (98.33%)	**<0.001**
	Yes	21 (0.58%)	43 (2.56%)		10 (0.24%)	21 (1.09%)		10 (0.23%)	23 (1.31%)		41 (0.35%)	87 (1.67%)	
Cancer	No	3335 (97.20%)	1535 (94.65%)	**<0.001**	3990 (98.78%)	1732 (97.09%)	**<0.001**	3661 (98.54%)	1752 (97.57%)	**0.009**	10,986 (98.17%)	5019 (96.41%)	**<0.001**
	Yes	94 (2.80%)	85 (5.35%)		52 (1.22%)	50 (2.91%)		63 (1.46%)	53 (2.43%)		209 (1.83%)	188 (3.59%)	
Migraine	No	3027 (88.23%)	1135 (69.87%)	**<0.001**	3744 (92.73%)	1350 (76.29%)	**<0.001**	3419 (90.93%)	1490 (79.69%)	**<0.001**	10,190 (90.63%)	3975 (75.23%)	**<0.001**
	Yes	402 (11.77%)	486 (30.13%)		299 (7.27%)	432 (23.71%)		317 (9.07%)	341 (20.31%)		1018 (9.37%)	1259 (24.77%)	
Depression	No	3312 (96.76%)	1406 (87.33%)	**<0.001**	3950 (97.68%)	1582 (89.16%)	**<0.001**	3630 (97.68%)	1670 (92.24%)	**<0.001**	10,892 (97.37%)	4658 (89.55%)	**<0.001**
	Yes	117 (3.24%)	215 (12.67%)		93 (2.32%)	200 (10.84%)		89 (2.32%)	139 (7.76%)		299 (2.63%)	554 (10.45%)	
Mental illness	No	3345 (97.61%)	1527 (94.45%)	**<0.001**	3965 (98.07%)	1677 (94.52%)	**<0.001**	3497 (94.34%)	1507 (84.03%)	**<0.001**	10,807 (96.70%)	4711 (91.06%)	**<0.001**
	Yes	84 (2.39%)	94 (5.55%)		78 (1.93%)	104 (5.48%)		206 (5.66%)	292 (15.97%)		368 (3.30%)	490 (8.94%)	
Hypercholesterolaemia	No	3158 (92.06%)	1243 (77.42%)	**<0.001**	3776 (93.54%)	1382 (78.06%)	**<0.001**	3330 (90.85%)	1344 (77.79%)	**<0.001**	10,264 (92.17%)	3969 (77.75%)	**<0.001**
	Yes	270 (7.94%)	370 (22.58%)		261 (6.46%)	392 (21.94%)		361 (9.15%)	417 (22.21%)		892 (7.83%)	1179 (22.25%)	
Arrhythmia	No	3152 (92.09%)	1160 (71.65%)	**<0.001**	3836 (95.07%)	1381 (78.36%)	**<0.001**	3491 (94.58%)	1444 (81.47%)	**<0.001**	10,479 (93.90%)	3985 (77.08%)	**<0.001**
	Yes	277 (7.91%)	460 (28.35%)		207 (4.93%)	400 (21.64%)		231 (5.42%)	362 (18.53%)		715 (6.10%)	1222 (22.92%)	
Other heart disease	No	3338 (97.28%)	1476 (91.69%)	**<0.001**	4003 (99.02%)	1703 (95.62%)	**<0.001**	3630 (97.89%)	1670 (94.09%)	**<0.001**	10,971 (98.06%)	4849 (93.77%)	**<0.001**
	Yes	90 (2.72%)	142 (8.31%)		40 (0.98%)	78 (4.38%)		94 (2.11%)	111 (5.91%)		224 (1.94%)	331 (6.23%)	
CVD	No	2973 (86.96%)	1007 (62.43%)	**<0.001**	3698 (91.56%)	1220 (69.65%)	**<0.001**	3337 (90.74%)	1314 (74.25%)	**<0.001**	10,008 (89.75%)	3541 (68.71%)	**<0.001**
	Yes	456 (13.04%)	614 (37.57%)		345 (8.44%)	562 (30.35%)		406 (9.26%)	518 (25.75%)		1207 (10.25%)	1694 (31.29%)	

Bold values indicate statistical significance (*p* < 0.05) based on weighted Pearson’s chi-squared test. Values are presented as weighted column percentages; counts are unweighted. Abbreviations: AMI, acute myocardial infarction; CAD, coronary artery disease; CVD, cardiovascular disease.

**Table 4:** Logistic regression results for factors associated with chronic back pain.

Characteristics		Chronic Back Pain			
		2009	2014	2019	Pooled
		Adjusted OR [95% CI]	Adjusted OR [95% CI]	Adjusted OR [95% CI]	Adjusted OR [95% CI]
Sex	Male	ref	ref	ref	ref
	Female	**0.72 [0.60–0.86]**	**0.82 [0.70–0.96]**	0.99 [0.85–1.17]	**0.85 [0.77–0.93]**
Age categories	65+	ref	ref	ref	ref
	15 to 34	**0.55 [0.39–0.77]**	**0.59 [0.44–0.78]**	0.77 [0.57–1.04]	**0.61 [0.51–0.72]**
	35 to 64	1.02 [0.79–1.31]	**0.76 [0.61–0.94]**	1.19 [0.94–1.51]	0.94 [0.82–1.08]
Relationship status	Single	ref	ref	ref	ref
	In a relationship	**1.21 [1.00–1.46]**	**1.28 [1.09–1.52]**	**1.38 [1.17–1.62]**	**1.26 [1.14–1.38]**
Education	Primary	ref	ref	ref	ref
	Secondary	1.06 [0.87–1.30]	0.84 [0.70–1.00]	0.90 [0.75–1.08]	0.93 [0.84–1.04]
	Tertiary	0.78 [0.58–1.04]	**0.73 [0.58–0.92]**	0.88 [0.69–1.12]	**0.84 [0.72–0.96]**
Employment	Unemployed	ref	ref	ref	ref
	Employed	1.16 [0.94–1.42]	1.10 [0.91–1.32]	0.87 [0.70–1.07]	1.05 [0.94–1.18]
Area of residence	Rural	ref	ref	ref	ref
	Urban	0.91 [0.76–1.10]	0.92 [0.78–1.08]	1.04 [0.88–1.24]	0.95 [0.86–1.05]
Region 7 cat	Central-Hungary	ref	ref	ref	ref
	Southern Great Plain	**1.36 [1.03–1.78]**	1.11 [0.87–1.42]	0.95 [0.73–1.25]	1.12 [0.96–1.30]
	Southern Transdanubia	1.20 [0.88–1.64]	1.15 [0.86–1.52]	1.02 [0.77–1.36]	1.11 [0.94–1.32]
	Northern Great Plain	1.08 [0.82–1.42]	1.04 [0.83–1.31]	1.25 [0.99–1.58]	1.10 [0.96–1.27]
	Central Transdanubia	1.09 [0.81–1.47]	0.92 [0.71–1.20]	1.00 [0.76–1.30]	0.98 [0.84–1.15]
	Northern Hungary	0.93 [0.70–1.24]	0.97 [0.74–1.25]	1.11 [0.85–1.45]	0.99 [0.85–1.15]
	Western Transdanubia	1.15 [0.86–1.53]	0.87 [0.66–1.15]	1.04 [0.79–1.36]	1.00 [0.85–1.17]
Financial status	Average	ref	ref	ref	ref
	Good	0.90 [0.68–1.19]	0.84 [0.68–1.04]	**0.83 [0.69–1.00]**	0.89 [0.79–1.01]
	Bad	**1.30 [1.07–1.58]**	1.14 [0.94–1.38]	0.97 [0.75–1.26]	**1.15 [1.02–1.29]**
Income quintiles	First	ref	ref	ref	ref
	Second	0.91 [0.70–1.17]	0.81 [0.64–1.03]	1.23 [0.97–1.56]	0.95 [0.83–1.09]
	Third	0.86 [0.66–1.13]	**0.78 [0.61–1.00]**	**1.38 [1.07–1.78]**	0.95 [0.83–1.10]
	Fourth	0.90 [0.68–1.19]	0.84 [0.64–1.10]	1.28 [0.99–1.66]	0.95 [0.82–1.09]
	Fifth	0.91 [0.66–1.24]	0.92 [0.68–1.23]	**1.64 [1.20–2.23]**	1.03 [0.87–1.22]
BMI	Overweight/Obese	ref	ref	ref	ref
	Normal	0.85 [0.70–1.02]	0.86 [0.73–1.01]	0.85 [0.71–1.00]	**0.85 [0.77–0.93]**
Smoking	Smoker	ref	ref	ref	ref
	Non-smoker	0.87 [0.72–1.04]	**0.74 [0.63–0.88]**	0.85 [0.71–1.02]	**0.81 [0.73–0.90]**
Alcohol	Drinker	ref	ref	ref	ref
	Abstinent	**0.80 [0.67–0.95]**	0.95 [0.81–1.13]	**0.82 [0.69–0.98]**	**0.84 [0.76–0.93]**
Self-perceived health	Average	ref	ref	ref	ref
	Good	**0.54 [0.44–0.66]**	**0.51 [0.42–0.61]**	**0.55 [0.45–0.67]**	**0.53 [0.48–0.60]**
	Bad	**1.42 [1.08–1.88]**	1.19 [0.91–1.55]	1.27 [0.98–1.66]	**1.29 [1.11–1.51]**
Last dental visit	More than a year ago	ref	ref	ref	ref
	Within the last year	1.09 [0.92–1.31]	**1.18 [1.01–1.37]**	0.95 [0.80–1.11]	1.08 [0.98–1.19]
Taking prescription drugs	No	ref	ref	ref	ref
	Yes	1.13 [0.91–1.41]	1.06 [0.87–1.29]	**1.32 [1.06–1.63]**	**1.16 [1.03–1.30]**
Taking OTC drugs or supplements	No	ref	ref	ref	ref
	Yes	**1.36 [1.13–1.63]**	**1.23 [1.06–1.43]**	**1.26 [1.03–1.53]**	**1.31 [1.20–1.44]**
Asthma	No	ref	ref	ref	ref
	Yes	0.94 [0.64–1.38]	0.94 [0.66–1.32]	1.00 [0.68–1.46]	0.97 [0.78–1.19]
Bronchitis	No	ref	ref	ref	ref
	Yes	1.22 [0.85–1.76]	**1.74 [1.23–2.46]**	1.18 [0.79–1.75]	**1.30 [1.05–1.61]**
AMI	No	ref	ref	ref	ref
	Yes	0.67 [0.44–1.03]	1.20 [0.74–1.93]	1.26 [0.80–1.99]	0.91 [0.70–1.18]
CAD	No	ref	ref	ref	ref
	Yes	1.04 [0.73–1.49]	0.95 [0.66–1.36]	0.92 [0.61–1.39]	0.94 [0.76–1.16]
Stroke	No	ref	ref	ref	ref
	Yes	**0.57 [0.35–0.92]**	0.75 [0.49–1.13]	0.76 [0.46–1.25]	**0.69 [0.53–0.90]**
Arrhythmia	No	ref	ref	ref	ref
	Yes	**1.32 [1.00–1.73]**	**1.39 [1.07–1.81]**	**1.63 [1.25–2.13]**	**1.40 [1.20–1.63]**
Other heart disease	No	ref	ref	ref	ref
	Yes	0.86 [0.56–1.33]	1.36 [0.78–2.40]	1.04 [0.69–1.58]	1.00 [0.77–1.29]
Hypertension	No	ref	ref	ref	ref
	Yes	**1.28 [1.03–1.59]**	1.21 [0.99–1.47]	1.09 [0.90–1.33]	**1.18 [1.05–1.32]**
Hypercholesterolaemia	No	ref	ref	ref	ref
	Yes	1.10 [0.85–1.44]	1.18 [0.93–1.49]	1.19 [0.95–1.50]	**1.15 [1.01–1.33]**
Diabetes	No	ref	ref	ref	ref
	Yes	0.84 [0.61–1.15]	1.00 [0.77–1.30]	0.83 [0.64–1.07]	0.90 [0.77–1.05]
Arthrosis	No	ref	ref	ref	ref
	Yes	**3.61 [2.94–4.42]**	**2.92 [2.40–3.54]**	**2.25 [1.85–2.73]**	**2.89 [2.59–3.24]**
Chronic neck pain	No	ref	ref	ref	ref
	Yes	**5.98 [4.70–7.61]**	**5.43 [4.34–6.79]**	**5.10 [4.06–6.41]**	**5.46 [4.78–6.23]**
Allergy	No	ref	ref	ref	ref
	Yes	**1.35 [1.07–1.71]**	**1.40 [1.13–1.72]**	**1.39 [1.14–1.70]**	**1.38 [1.22–1.56]**
Peptic ulcer	No	ref	ref	ref	ref
	Yes	**1.72 [1.27–2.32]**	1.32 [0.87–2.03]	0.97 [0.62–1.53]	**1.40 [1.13–1.73]**
Chronic liver disease	No	ref	ref	ref	ref
	Yes	0.93 [0.46–1.87]	1.42 [0.62–3.26]	2.30 [0.92–5.75]	1.23 [0.77–1.96]
Cancer	No	ref	ref	ref	ref
	Yes	**0.58 [0.37–0.88]**	0.78 [0.47–1.30]	0.93 [0.56–1.55]	**0.67 [0.51–0.88]**
Migraine	No	ref	ref	ref	ref
	Yes	**1.73 [1.38–2.17]**	**2.23 [1.78–2.80]**	**2.31 [1.82–2.95]**	**2.02 [1.77–2.31]**
Depression	No	ref	ref	ref	ref
	Yes	1.04 [0.74–1.48]	0.98 [0.68–1.41]	1.13 [0.74–1.73]	1.02 [0.82–1.26]
Mental illness	No	ref	ref	ref	ref
	Yes	0.77 [0.46–1.28]	0.90 [0.56–1.45]	**1.53 [1.14–2.06]**	1.18 [0.95–1.48]

Adjusted odds ratios (ORs) with 95% confidence intervals (CIs) are derived from multiple logistic regression models including all covariates shown. Bold values indicate statistical significance (*p* < 0.05).

## Data Availability

The data are available upon request.
